# Recovery of facial expressions using functional electrical stimulation after full-face transplantation

**DOI:** 10.1186/s12984-018-0356-0

**Published:** 2018-03-06

**Authors:** Çağdaş Topçu, Hilmi Uysal, Ömer Özkan, Özlenen Özkan, Övünç Polat, Merve Bedeloğlu, Arzu Akgül, Ela Naz Döğer, Refik Sever, Ömer Halil Çolak

**Affiliations:** 1 Faculty of Engineering, Department of Electrical-Electronics Engineering, Akdeniz University, Dumlupınar Bulv. 07058 Campus, Antalya, Turkey; 2 Institute of Physiology, Medical University of Graz, Harrachgasse 21/5, 8010, Graz, Austria; 3 Faculty of Medicine, Department of Neurology, Akdeniz University, Antalya, Turkey; 4 Faculty of Medicine, Department of Plastic and Reconstructive Surgery, Akdeniz University, Antalya, Turkey

**Keywords:** Full-face transplantation, emotional expressions, rehabilitation, functional electrical stimulation, surface electromyography, fuzzy entropy, complexity

## Abstract

**Background:**

We assessed the recovery of 2 face transplantation patients with measures of complexity during neuromuscular rehabilitation. Cognitive rehabilitation methods and functional electrical stimulation were used to improve facial emotional expressions of full-face transplantation patients for 5 months. Rehabilitation and analyses were conducted at approximately 3 years after full facial transplantation in the patient group. We report complexity analysis of surface electromyography signals of these two patients in comparison to the results of 10 healthy individuals.

**Methods:**

Facial surface electromyography data were collected during 6 basic emotional expressions and 4 primary facial movements from 2 full-face transplantation patients and 10 healthy individuals to determine a strategy of functional electrical stimulation and understand the mechanisms of rehabilitation. A new personalized rehabilitation technique was developed using the wavelet packet method. Rehabilitation sessions were applied twice a month for 5 months. Subsequently, motor and functional progress was assessed by comparing the fuzzy entropy of surface electromyography data against the results obtained from patients before rehabilitation and the mean results obtained from 10 healthy subjects.

**Results:**

At the end of personalized rehabilitation, the patient group showed improvements in their facial symmetry and their ability to perform basic facial expressions and primary facial movements. Similarity in the pattern of fuzzy entropy for facial expressions between the patient group and healthy individuals increased. Synkinesis was detected during primary facial movements in the patient group, and one patient showed synkinesis during the happiness expression. Synkinesis in the lower face region of one of the patients was eliminated for the lid tightening movement.

**Conclusions:**

The recovery of emotional expressions after personalized rehabilitation was satisfactory to the patients. The assessment with complexity analysis of sEMG data can be used for developing new neurorehabilitation techniques and detecting synkinesis after full-face transplantation.

**Electronic supplementary material:**

The online version of this article (10.1186/s12984-018-0356-0) contains supplementary material, which is available to authorized users.

## Background

Understanding the rehabilitation of facial muscles and nerves after facial transplantation has remained a challenging problem in neurorehabilitation [1–8] since the first partial face transplantation was performed in 2005 [9]. Recovery of facial expressions improves the nonverbal communication and social interactions of patients; consequently, it can be considered as an integral part of the rehabilitation of patients after transplantation. Since 2005, 37 partial and full-face transplantations have been performed worldwide [5, 10–15]. The ability to perform facial expressions improved in 76% of the reported 24 facial transplantation recipients worldwide [3]. Face transplantation still cannot be considered an ordinary surgical procedure [16]. Thus, face transplantation patients generally have different backgrounds and exhibit different degrees of motor and sensory improvements; therefore, each case needs to be treated individually. The complexity of facial nerves and randomness of reinnervation may be responsible for unexpected observations of aberrant reinnervation and synkinesis after facial transplantation [1], the latter of which is also observed in peripheral facial palsy and hemifacial spasm patients [17, 18]. In addition, the nature of involuntary eyelid blink restoration is a complicated process, and reinnervation is unpredictable [4].

Functional electrical stimulation is a noninvasive technique that is used for rehabilitation of patients after stroke [19], spinal cord injury [20] or neurodegenerative diseases [21]. Investigation of the effects of this method on rehabilitation after full-face transplantation is an important step towards understanding recovery process.

We propose a new individualized rehabilitation method with selective FES for patients after full-face transplantation and an assessment of the results of rehabilitation by comparing complexity measures from multichannel sEMG data. Neuromuscular stimulation modality was enhanced with visual stimuli to improve the cognitive aspects of the rehabilitation process. We chose a fuzzy entropy based method as a complexity measure; this measure was used to analyze sEMG data in various ways [22–26] and to assess robot assisted rehabilitation training of patients after stroke [27].

## Methods

### Individualized neurorehabilitation

Functional electrical stimulation was performed on both transplantation patients during primary facial and facial expression movements to improve these movements. A battery powered 8-channel electrical stimulator with a peak amplitude of 10 mA was used (RehaStim-1, Hasomed GmbH). The frequency and the pulse width of the stimulation were fixed as 30 Hz and 60 μs, respectively. The duration of the stimulation was 40 s for each movement in a session. Facial sEMG signals (sample rate, fs = 2000 Hz) collected from healthy individuals and patients were used to investigate active muscles in each movement. The selected healthy individuals were male (31 ± 5 years). Fourteen bipolar electrodes (9-mm diameter and 20-mm interelectrode distance) were used to collect sEMG data and apply electrical stimulations. The electrode locations of the measurements are described in the Supplementary Material to Ref. [1]. Subjects were asked to perform simple facial expressions (angry, fearful, happy, hateful, surprised, and sad) and primary facial movements (lid tightening, lip funneling, lip puckering, and outer brow raising) 4 times during each recording. Wavelet packet transforms were performed on the collected data, and the mean value of wavelet packet energy was calculated for 10 healthy individuals (for more detail, see Topçu et al. [1]). These mean values for each sEMG channel were compared with transplants’ results, and thus, differences in activation places were determined for each transplantation patient (Supplementary material, Additional file 1, Table S1, Table S2). Thus, different facial movements were selectively supported by functional electrical stimulation [28, 29]. A healthy individual performed the same movements with patients concurrently to develop the cognitive aspect of the rehabilitation process; additionally, pictures of the emotional expressions and a live video of the patients’ own faces were shown simultaneously to the patients during electrical stimulations and facial sEMG recordings to improve the same aspect of the rehabilitation method. Rehabilitation sessions were conducted twice a month for 5 months.

Semmes-Weinstein’s monofilament test (SWMT) was used to investigate sensory innervation.

### Patient A

A 37-year-old male had lost his facial muscles in a burn accident when he was 3 years old. He was not able to perform facial expressions when he was admitted to the polyclinic. He underwent full-face transplantation in May 2012. All muscles for facial expression and eyelids were transplanted, but those for mastication were not [1]. His evoked facial expression videos were shown to healthy individuals, and his emotional expressions from the most to least recognizable were happiness, surprise, anger, fear, sadness, and disgust [6]. Rehabilitation sessions were conducted 33 months after transplantation.

### Patient B

Patient B was a 22-year-old male who had burns and lost his facial muscles due to exposure to boiling water at 7 months of age. He was also not able to perform facial expressions when he was admitted to our plastic and reconstructive surgery polyclinic. He underwent face transplantation (excluding eyelids) in January 2012. The infraorbital, supraorbital, and mental nerves were coapted. The lower branches were also coapted to the donor’s facial nerve trunk [1]. He showed a phantom sensation phenomenon that was related to cortical plasticity [30]. Rehabilitation sessions were conducted 37 months after transplantation.

### Fuzzy entropy

W. Chen et al. developed fuzzy entropy (FuzzyEn) [22] to measure the complexity of sEMG signals as an improved version of sample entropy [31, 32]. Given a time series of data *u*(1), *u*(2), …, *u*(*N*) from the measurements, form a sequence of vectors *x*(1), *x*(2), …, *x*(*N* − *m* + 1) in *R*^*m*^ for the selected embedded dimension m, defined by *x*^*m*^(*i*) = [*u*(*i*), *u*(*i* + 1), …, *u*(*i* + *m* − 1)] for each *i*, 1 ≤ *i* ≤ *N* − *m* + 1. Then, the distance ${d}_{ij}^m$ of two vectors *x*^*m*^(*i*) and *x*^*m*^(*j*) should be the following:

1 $$ {d}_{ij}^m=\underset{k\in \left(0,m-1\right)}{\max}\left|u\left(i+k\right)-{u}_0(i)-u\left(j+k\right)+{u}_0(j)\right|. $$

A family of exponential functions is used as the fuzzy function, which measures the similarity degree of two vectors:

2 $$ {D}_{ij}^m=\mu \left({d}_{ij}^m,n,r\right)=\exp \left(-\frac{{\left({d}_{ij}^m\right)}^n}{r}\right), $$

where the parameter of the exponential is chosen as n = 2. The family of exponential functions is convex and continuous. The function *φ*^*m*^(*n*, *r*) is defined as follows:

3 $$ {\varphi}^m\left(n,r\right)=\frac{1}{N-m}\sum \limits_{i=1}^{N-m}\left(\frac{1}{N-m-1}\sum \limits_{\begin{array}{c}j=1\\ {}j\ne 1\end{array}}^{N-m}{D}_{ij}^m\right), $$

FuzzyEn parameter can be defined from the time series as follows:

4 $$ FuzzEn\left(m,n,r\right)=\underset{N\to \infty }{\lim}\left[\ln {\varphi}^m\left(n,r\right)-\ln {\varphi}^{m+1}\left(n,r\right)\right]. $$

The tolerance value r and embedded dimension m were fixed as *r* = 0.15 and *m* = 2, respectively [22, 27, 33].

## Results

Fuzzy entropy values were calculated for each movement for the patient group before and after rehabilitation. Fuzzy entropy values smaller than 0.01 were removed to clean the muscle resting state; subsequently, the mean values of fuzzy entropy measures were calculated for each channel to evaluate the effects of the rehabilitation sessions. The results are given for primary facial movements and simple facial expressions.

### Primary facial movements

Overall, we observed improvements in symmetry and similarity between the patient group and healthy individuals during the 4 primary facial movements after rehabilitation. Moreover, synkinesis was eliminated for the lid tightening movement by using our rehabilitation method.

**Figure 1:**
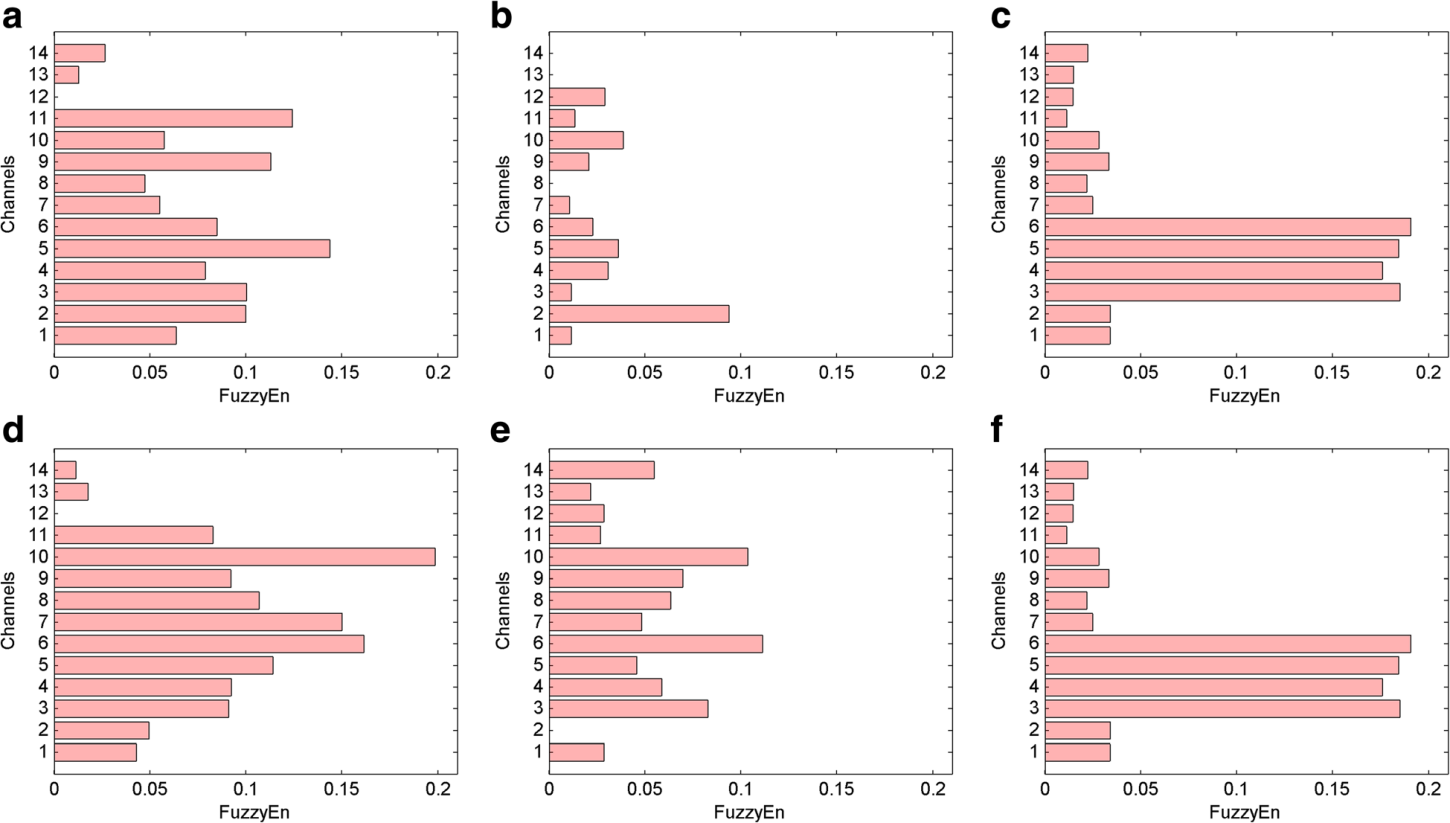
Facial muscle activity representations based on the FuzzyEn method for the lid tightening primary movement. **a** Estimated muscle activities of patient A before rehabilitation. **b** Estimated muscle activities of patient A after rehabilitation. **c**, **f** Mean value of estimated muscle activities of ten healthy individuals. **d** Estimated muscle activities of patient B before rehabilitation. **e** Estimated muscle activities of patient B after rehabilitation.

**Figure 2:**
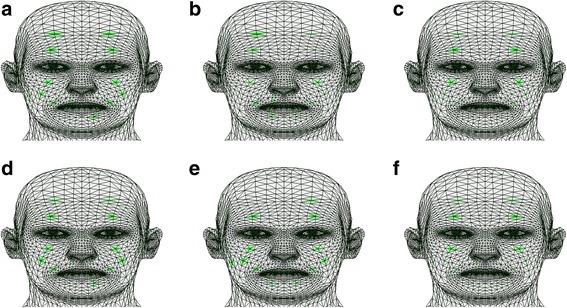
Facial muscle activity representations on a 3D face model based on the FuzzyEn method for the lid tightening primary movement. **a** Estimated muscle activities of patient A before rehabilitation. **b** Estimated muscle activities of patient A after rehabilitation. **c**, **f** Mean value of estimated muscle activities of ten healthy individuals. **d** Estimated muscle activities of patient B before rehabilitation. **e** Estimated muscle activities of patient B after rehabilitation.

For the lid tightening movement, synkinesis in the lower face of patient A was eliminated with rehabilitation. Similarity in the pattern of fuzzy entropy for this primary motion between patient A and healthy individuals increased after the rehabilitation process. Fuzzy entropy data for the lid tightening facial movement in all subjects are illustrated in Figure 1. Fuzzy entropy data for the lid tightening facial movement in all subjects are illustrated on a 3D face model in Figure 2.

For the lip funneling movement, the activity of the orbicularis oris muscle in the patient group became more symmetric, but the patient group showed synkinesis in their frontalis muscle, and this deficiency remained after the rehabilitation process.

For the lip puckering movement, the patient group showed more symmetric muscle activity in their lower face, but synkinesis remained in their upper face after rehabilitation.

For outer brow raising, patient A showed improvements in the left side of his upper face. Synkinesis remained in his lower face after rehabilitation. Patient B showed a similar pattern to that of healthy individuals before and after rehabilitation.

Patient A showed 1.4±0.1 between 19 and 39 months postoperatively and patient B showed 1.2±0.2 improvements between 23 and 43 months postoperatively according to SWMT (see supplementary material, Additional file 1, Table S4, and Table S5 for more details).

### Facial expressions

During the anger facial expression, patient A showed more symmetric muscle activity after neurorehabilitation. Patient B showed more balance in this expression, and the similarity in the pattern of fuzzy entropy for this emotional motion between patient B and healthy individuals increased after the rehabilitation process.

During the fear facial expression, patient A showed improvements in the lower part of his face, and his pattern of fuzzy entropy became more similar to the pattern of healthy individuals after the rehabilitation process. The activity of the depressor labii inferious muscle of patient B became more symmetric after functional electrical therapy.

**Figure 3:**
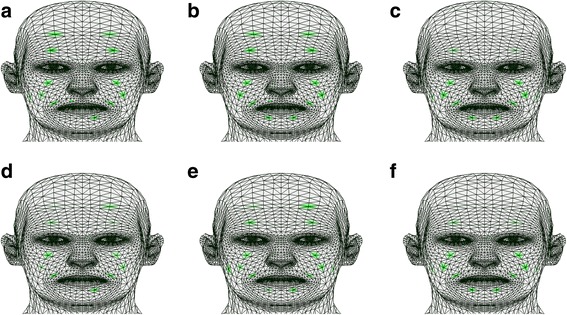
Facial muscle activity representations on a 3D face model based on the FuzzyEn method for the happiness facial expression. **a** Estimated muscle activities of patient A before rehabilitation. **b** Estimated muscle activities of patient A after rehabilitation. **c**, **f** Mean value of estimated muscle activities of ten healthy individuals. **d** Estimated muscle activities of patient B before rehabilitation. **e** Estimated muscle activities of patient B after rehabilitation.

During the happiness facial expression, both cases showed improvements in the lower face region after rehabilitation. Moreover, patient B showed a more similar pattern to that of healthy subjects. Synkinesis was detected in the frontalis muscle of patient B, and it was also observed after rehabilitation. Fuzzy entropy data for the happiness facial expression in all subjects are illustrated on a 3D face model in Figure 3.

During the hateful/disgust facial expression, patient A showed improvements in the lower face region after rehabilitation. The level of muscle activities increased and became more symmetric in patient B for this emotional expression after rehabilitation.

During the sadness facial expression, both cases showed more symmetric muscle activities in the lower face region after rehabilitation.

During the surprised facial expression, patient A showed a symmetric pattern after the rehabilitation process. Patient B showed improvements in the frontalis muscle region after rehabilitation.

The correlation coefficient of multichannel fuzzy entropy values for primary facial movements between the patient group and healthy individuals increased by a factor of 3.37 and the coefficient increased by a factor of 1.35 for facial expressions after rehabilitation. Although the patients showed improvements in primary facial movements satisfactorily, patients showed improvements in emotional expressions gradually during rehabilitation.

## Discussion

The change in motor, sensory and cognitive functions observed in the reinnervation process that develops after total denervation in full-face transplants is not yet fully understood. One of the main reasons for the “facial mask” phenomenon observed in full-face transplant individuals is undoubtedly that the reinnervation of facial muscles has not yet developed. However, can the differences observed in primary motor and sensory expressions after reinnervation be explained by only the decrease in innervation strength? Whether the changes that can be observed in cortical motor and sensory presentations are contributing to the question has not yet been answered. For the muscles of the reinnervation to emit emotional expressions, the appropriate cortical program needs to be effectively transferred to the periphery. However, we have findings that suggest that there are problems in this regard.

After 5 months using FES, the patient group showed improvements in mostly their lower face region and several facial muscle groups. Similarity in the pattern of fuzzy entropy for emotional motions between the patient group and healthy individuals and symmetry in muscle activity locations increased after the rehabilitation process. The personalized rehabilitation method was developed to particularly improve the facial expression abilities of our patients, and the results are satisfactory to the patient group. There are different rehabilitation methods [2] for this purpose, and some may help facial transplantation patients achieve reduced synkinesis [34, 35]. A full facial transplantation patient reported improvements in her smiling ability after an 8-week lip-closure exercise and motor program [7]. These improvements were confirmed not by electrophysiological methods but rather by an interview. Gradual improvements were observed in muscle movements using manual muscle testing of facial expressions, and they demonstrated the evolution of smiling ability of a face transplantation patient and synkinesis between 6 to 42 months with video records [36]. Fuzzy entropy of sEMG data can be combined with other rehabilitation assessment methods to collect more information about the rehabilitation process of face transplantation patients, and it will allow a more standardized assessment of functional and motor recovery.

Synkinesis was detected during all primary face movements, and synkinesis in the lower face of patient A was eliminated for the lid tightening movement with rehabilitation. During the happiness facial expression, patient B showed synkinesis in his frontalis muscle. Facial nerve axons start to grow and remake neuromuscular junctions with muscles 3 months after axonal damage, and aberrant reinnervation can possibly occur during nerve recovery [17]; moreover, facial nerve recovery of a facial transplantation patient was observed with sEMG at 1 month post-operatively [37]. Thus, the treatment of facial synkinesis could begin at the early stage of the post-operative phase of face transplantation. A live video of the patients’ own faces was shown simultaneously to the patients during electrical stimulations and facial sEMG recordings, and this biofeedback could help eliminate synkinesis [38]. However, inappropriate usage of electrical stimulation may increase synkinesis [2]. On the other hand, synkinesis can be minimized with minimization of the distance between facial nerve coaptation and target muscles [36].

When comparing the success of primary facial movements before and after FES to that of emotional movements before and after FES, the success of emotional movements is more evident, which suggests that rehabilitation is important and that there may be changes in the cortical representation of facial emotional movements in facial transplant cases. We think that motor programs related to emotions are influenced before the transfer and that there are problems in transferring the universal motor program to the periphery after the transfer. The fact that disorders such as those associated with face sensation have been shown is also a feature of this result [6, 8].

Improvements in primary facial movements and facial expressions are not possible without reinnervation. Our method basically evaluates improvements based on reinnervation. Thus, although the reinnervation occurs we showed deficiencies of expressing facial emotions and we suggested rehabilitation methods which have cognitive aspects for this problem. Sole effects of reinnervation without rehabilitation could be assessed with control cases but with very few cases it was impossible to recognize and that issue is our limit and weakness of this study.

## Conclusions

In this study, we developed and tested a personalized multichannel FES rehabilitation technique for the rehabilitation of facial expressions after full-face transplantation. The rehabilitation method was individualized based on the muscle activity locations of each patient when they performed different facial movements. During rehabilitation sessions, a healthy individual synchronously performed the same facial expression movements with each patient to develop a cognitive connection between their facial expressions and their social interactions. Complexity analysis of sEMG was used to assess motor and functional improvements during the personalized rehabilitation method, and it demonstrated that the symmetry of facial muscle activity and similarity in the pattern of fuzzy entropy for emotional motions between the patient group and healthy individuals increased. Additionally, the rehabilitation method could be used for functional movement rehabilitation of patients with partial face transplantation, arm transplantation and replantation, and stroke survivors.

## Additional file

Additional file 1: Recovery of facial expressions using functional electrical stimulation after full-face transplantation. (DOCX 6090 kb)

## Abbreviations

FES: Functional electrical stimulation
FuzzyEn: Fuzzy entropy
sEMG: Surface electromyography
SWMT: Semmes-Weinstein’s monofilament test
