# Supplementary material for: Recovery of facial expressions using functional electrical stimulation after full-face transplantation
Source: J Neuroeng Rehabil. 2018 Mar 6;15:15. doi: 10.1186/s12984-018-0356-0 (PMC5840782; doi:10.1186/s12984-018-0356-0)
Supplement: Supplementary file 1 — Recovery of facial expressions using functional electrical stimulation after full-face transplantation. (DOCX 6090 kb) [file 12984_2018_356_MOESM1_ESM.docx]

**Recovery of facial expressions using functional electrical stimulation**

**after full-face transplantation**

**Supplementary Material**

Çağdaş Topçu MSc^1,2^, Hilmi Uysal MD^3^, Ömer Özkan MD^4^, Özlenen Özkan MD^4^,

Övünç Polat PhD^1^, Merve Bedeloğlu MSc^1^, Arzu Akgül MSc^1^, Ela Naz Döğer BSc^1^,

Refik Sever PhD^1^, Ömer Halil Çolak PhD^1^

^1^Akdeniz University, Faculty of Engineering, Department of Electrical-Electronics Engineering

^2^Medical University of Graz, Institute of Physiology

^3^Akdeniz University, Faculty of Medicine, Department of Neurology

^4^Akdeniz University, Faculty of Medicine, Department of Plastic and Reconstructive Surgery

Corresponding author:

Çağdaş Topçu

Faculty of Engineering, Department of Electrical-Electronics Engineering, Akdeniz University

Dumlupınar Bulv. 07058 Campus Antalya, Turkey

Institute of Physiology, Medical University of Graz

Harrachgasse 21/5, 8010 Graz, Austria

Phone: +43 676 5758329

E-mail: [cagdas.topcu@medunigraz.at](mailto:cagdas.topcu@medunigraz.at)

Çağdaş Topçu [cagdas.topcu@medunigraz.at](mailto:cagdas.topcu@medunigraz.at)

Hilmi Uysal [uysalh@akdeniz.edu.tr](mailto:uysalh@akdeniz.edu.tr)

Ömer Özkan [omozkan@hotmail.com](mailto:omozkan@hotmail.com)

Özlenen Özkan [ozlenend@yahoo.com](mailto:ozlenend@yahoo.com)

Övünç Polat [ovuncpolat@akdeniz.edu.tr](mailto:ovuncpolat@akdeniz.edu.tr)

Merve Bedeloğlu [mrvbedeloglu@gmail.com](mailto:mrvbedeloglu@gmail.com)

Arzu Akgül [arzuakgul@akdeniz.edu.tr](mailto:arzuakgul@akdeniz.edu.tr)

Ela Naz Döğer [elanazdoger@gmail.com](mailto:elanazdoger@gmail.com)

Refik Sever [refiksever@gmail.com](mailto:refiksever@gmail.com)

Ömer Halil Çolak [omercol@akdeniz.edu.tr](mailto:omercol@akdeniz.edu.tr)

**Figure S1:** Facial muscle activity representations based on the FuzzyEn method for the lip funneling facial movement. A) Estimated muscle activities of patient A before rehabilitation. B) Estimated muscle activities of patient A after rehabilitation. C, F) Mean value of estimated muscle activities of ten healthy individuals. D) Estimated muscle activities of patient B before rehabilitation. E) Estimated muscle activities of patient B after rehabilitation.


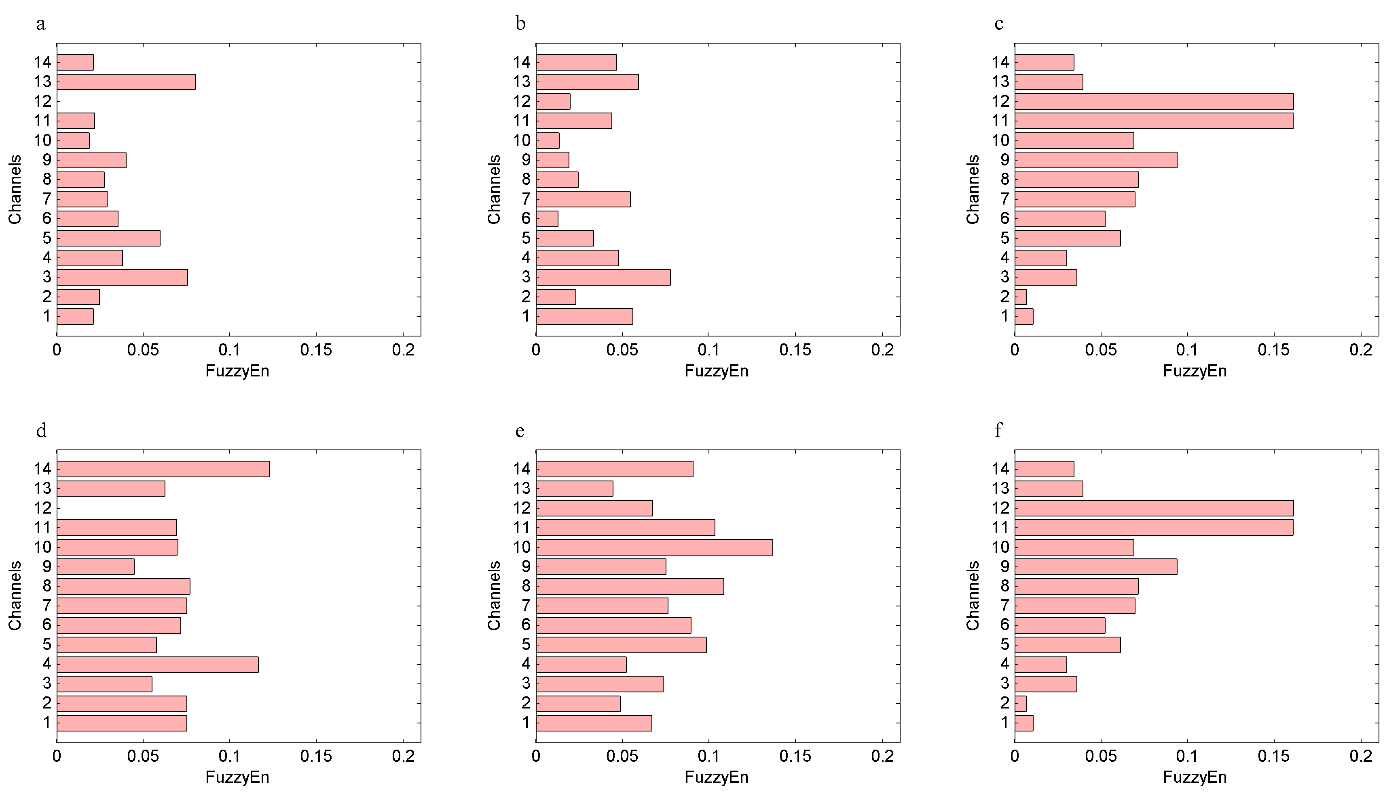


**Figure S2:** Facial muscle activity representations on a 3D face model based on the FuzzyEn method for the lip funneling facial movement. A) Estimated muscle activities of patient A before rehabilitation. B) Estimated muscle activities of patient A after rehabilitation. C, F) Mean value of estimated muscle activities of ten healthy individuals. D) Estimated muscle activities of patient B before rehabilitation. E) Estimated muscle activities of patient B after rehabilitation.
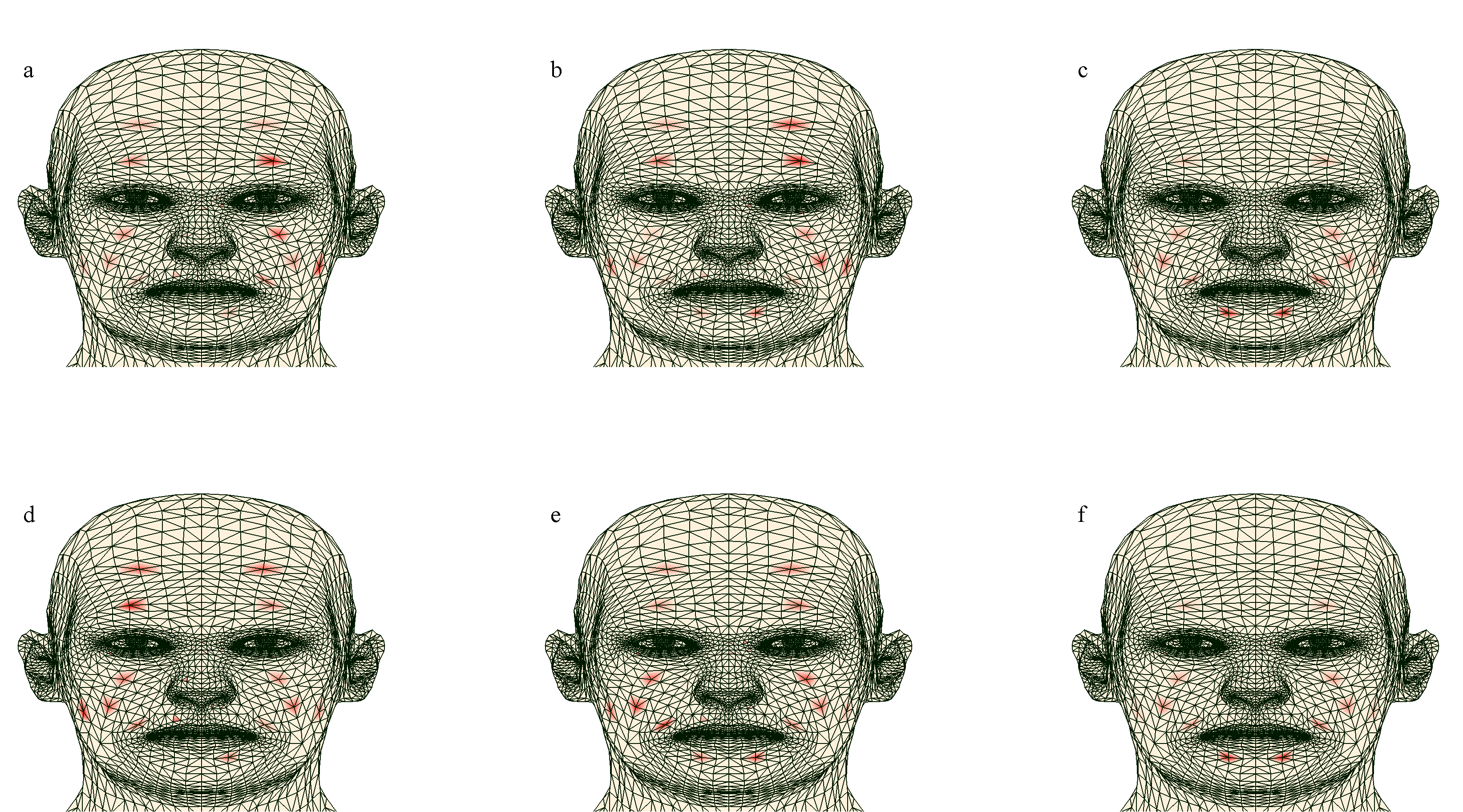


**Figure S3:** Facial muscle activity representations based on the FuzzyEn method for the lip puckering facial movement. A) Estimated muscle activities of patient A before rehabilitation. B) Estimated muscle activities of patient A after rehabilitation. C, F) Mean value of estimated muscle activities of ten healthy individuals. D) Estimated muscle activities of patient B before rehabilitation. E) Estimated muscle activities of patient B after rehabilitation.


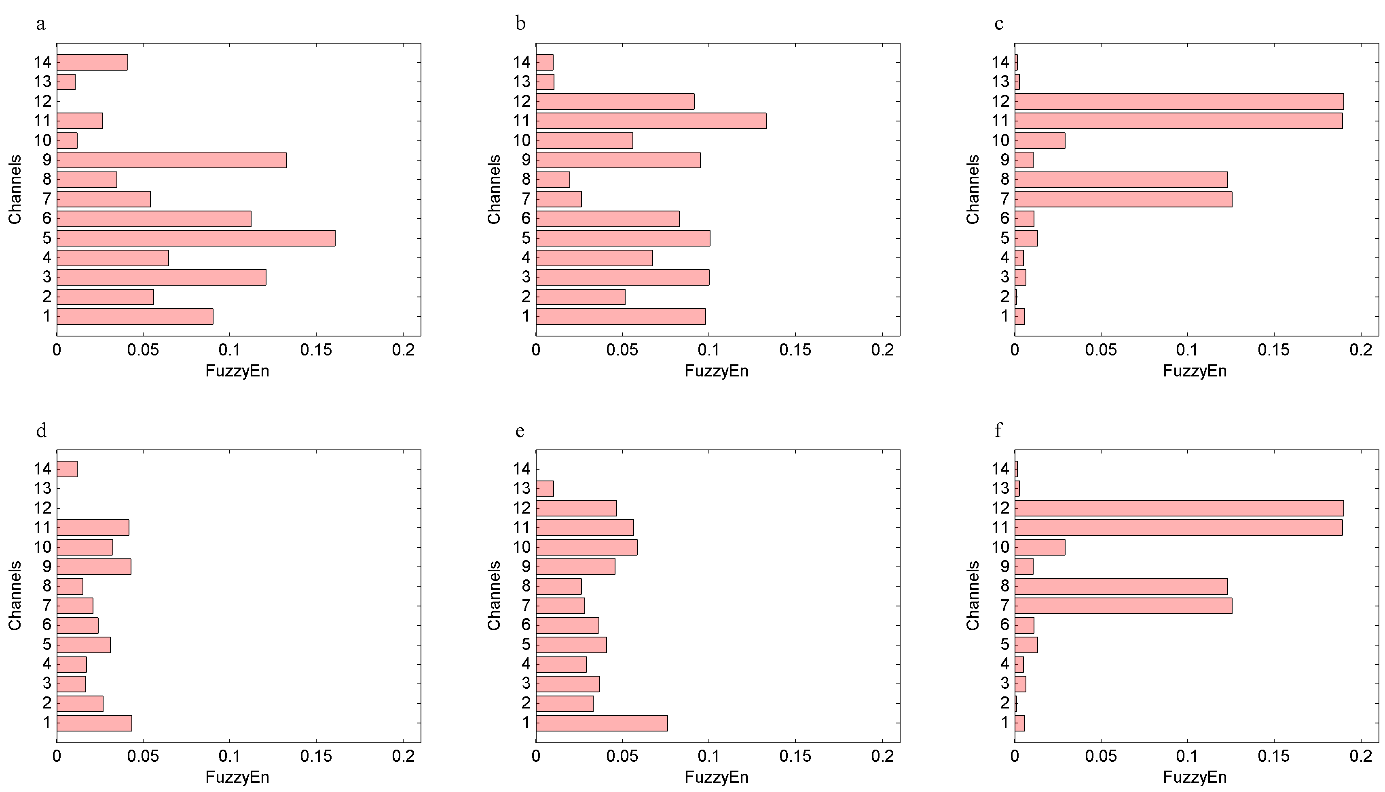


**Figure S4:** Facial muscle activity representations on a 3D face model based on the FuzzyEn method for the lip puckering facial movement. A) Estimated muscle activities of patient A before rehabilitation. B) Estimated muscle activities of patient A after rehabilitation. C, F) Mean value of estimated muscle activities of ten healthy individuals. D) Estimated muscle activities of patient B before rehabilitation. E) Estimated muscle activities of patient B after rehabilitation.


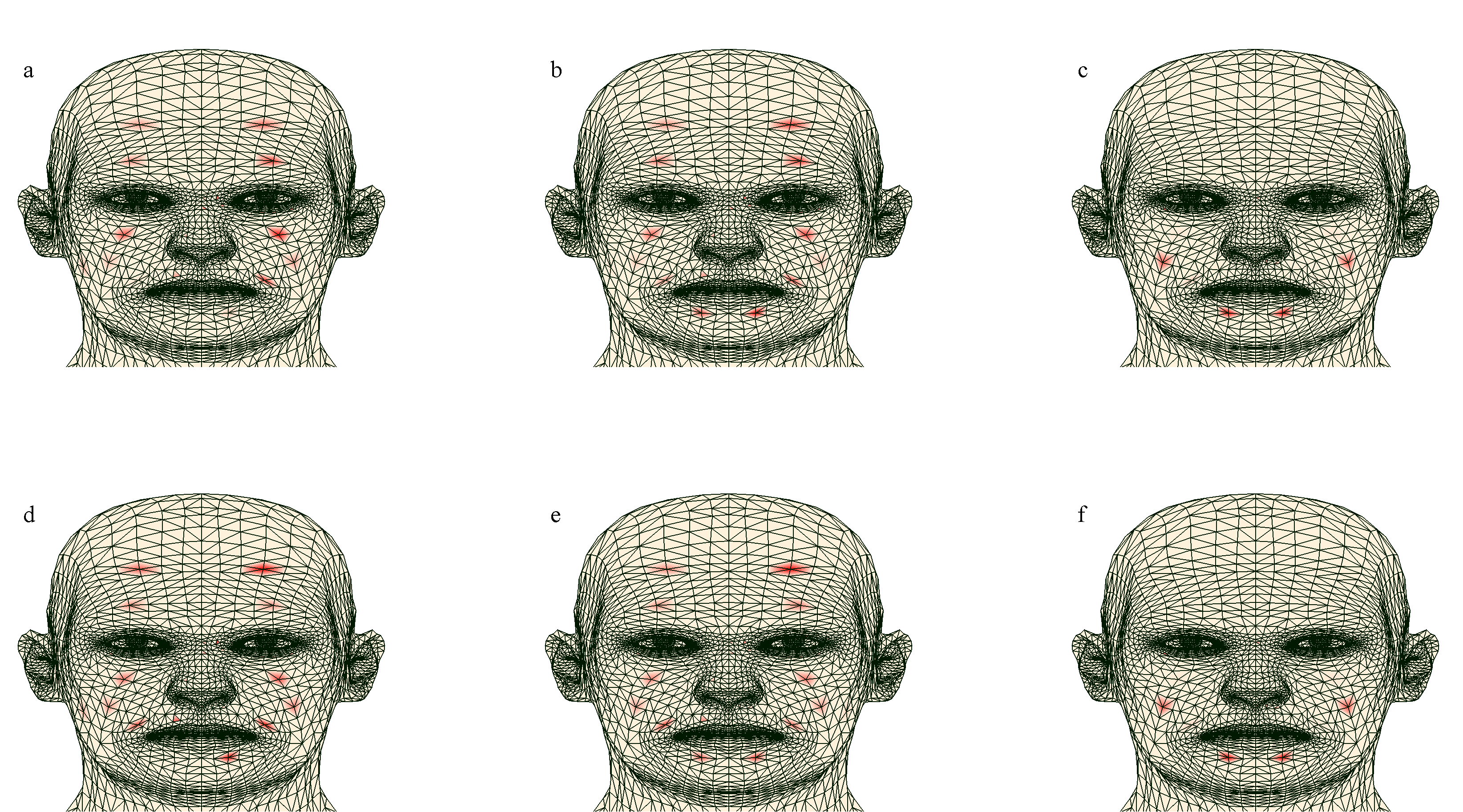


**Figure S5:** Facial muscle activity representations based on the FuzzyEn method for the outer brow raising facial movement. A) Estimated muscle activities of patient A before rehabilitation. B) Estimated muscle activities of patient A after rehabilitation. C, F) Mean value of estimated muscle activities of ten healthy individuals. D) Estimated muscle activities of patient B before rehabilitation. E) Estimated muscle activities of patient B after rehabilitation.


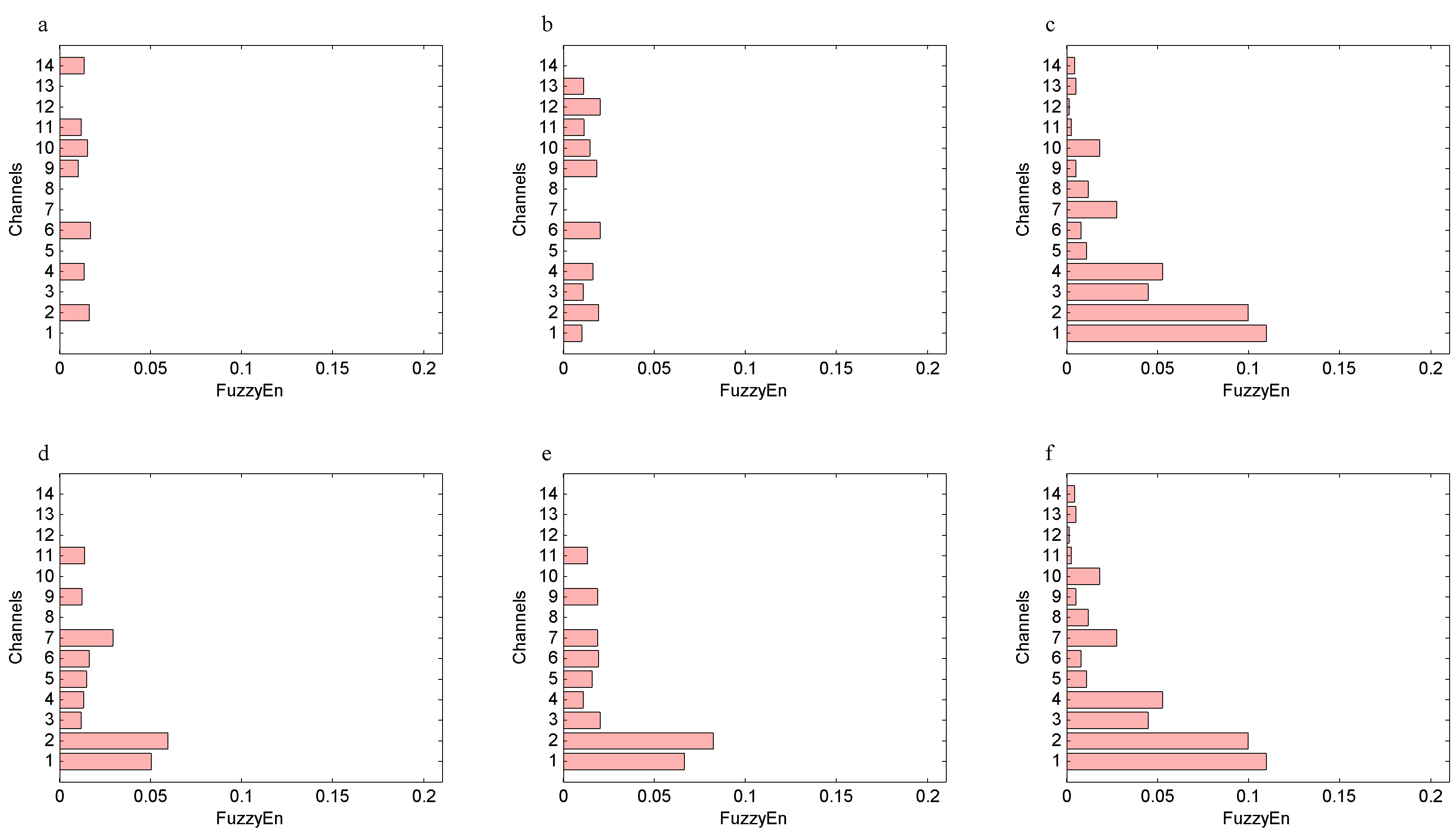


**Figure S6:** Facial muscle activity representations on a 3D face model based on the FuzzyEn method for the outer brow raising facial movement. A) Estimated muscle activities of patient A before rehabilitation. B) Estimated muscle activities of patient A after rehabilitation. C, F) Mean value of estimated muscle activities of ten healthy individuals. D) Estimated muscle activities of patient B before rehabilitation. E) Estimated muscle activities of patient B after rehabilitation.


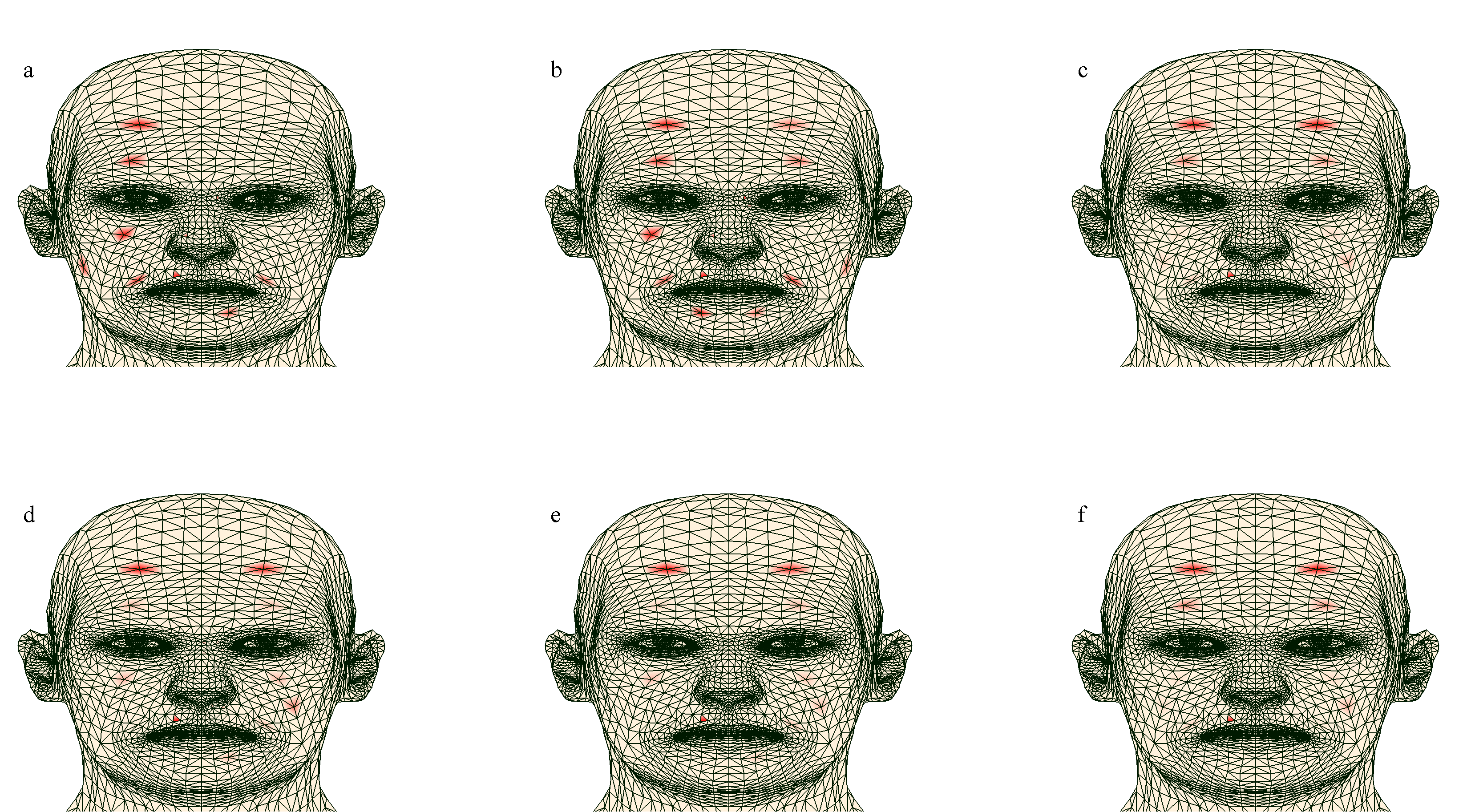


**Figure S7:** Facial muscle activity representations based on the FuzzyEn method for the anger facial expression. A) Estimated muscle activities of patient A before rehabilitation. B) Estimated muscle activities of patient A after rehabilitation. C, F) Mean value of estimated muscle activities of ten healthy individuals. D) Estimated muscle activities of patient B before rehabilitation. E) Estimated muscle activities of patient B after rehabilitation.


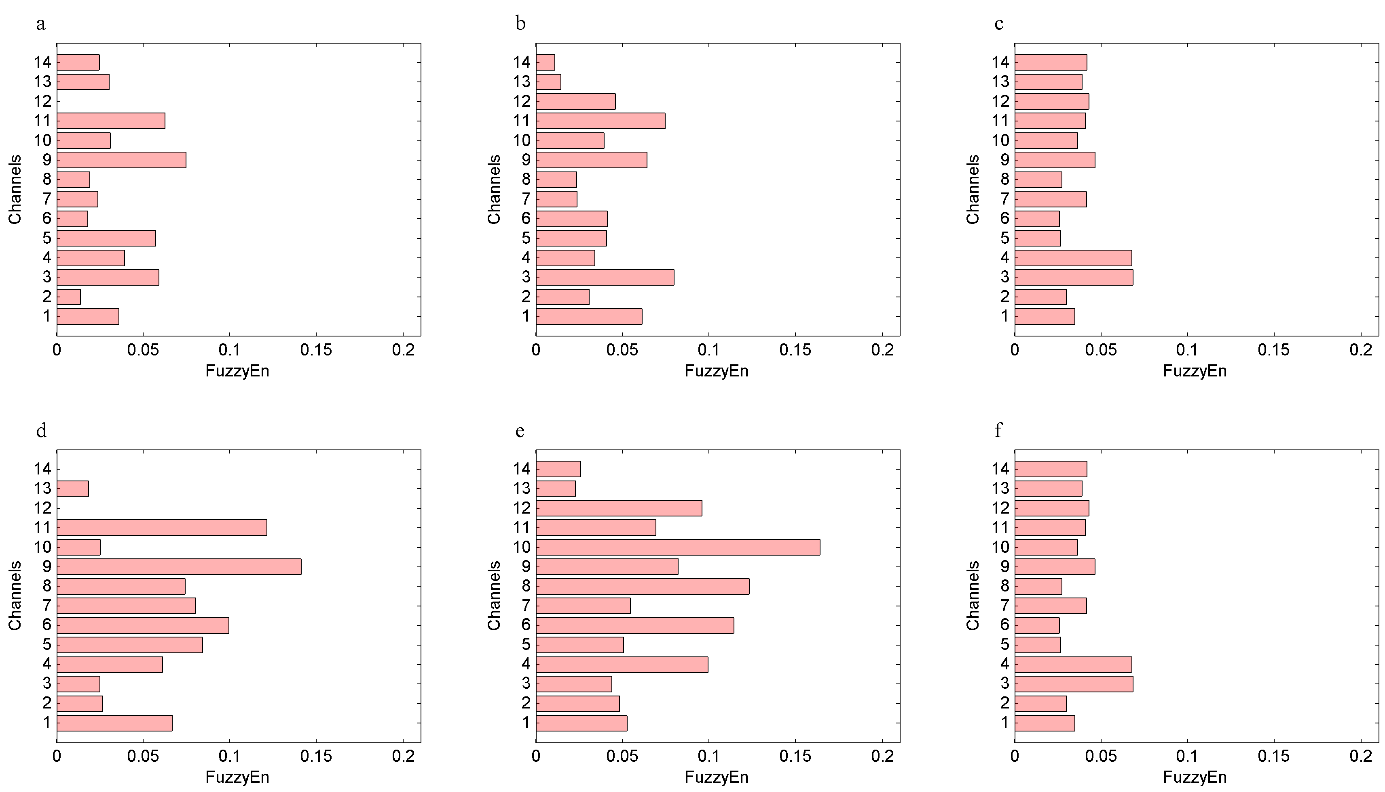


**Figure S8:** Facial muscle activity representations on a 3D face model based on the FuzzyEn method for the anger facial expression. A) Estimated muscle activities of patient A before rehabilitation. B) Estimated muscle activities of patient A after rehabilitation. C, F) Mean value of estimated muscle activities of ten healthy individuals. D) Estimated muscle activities of patient B before rehabilitation. E) Estimated muscle activities of patient B after rehabilitation.


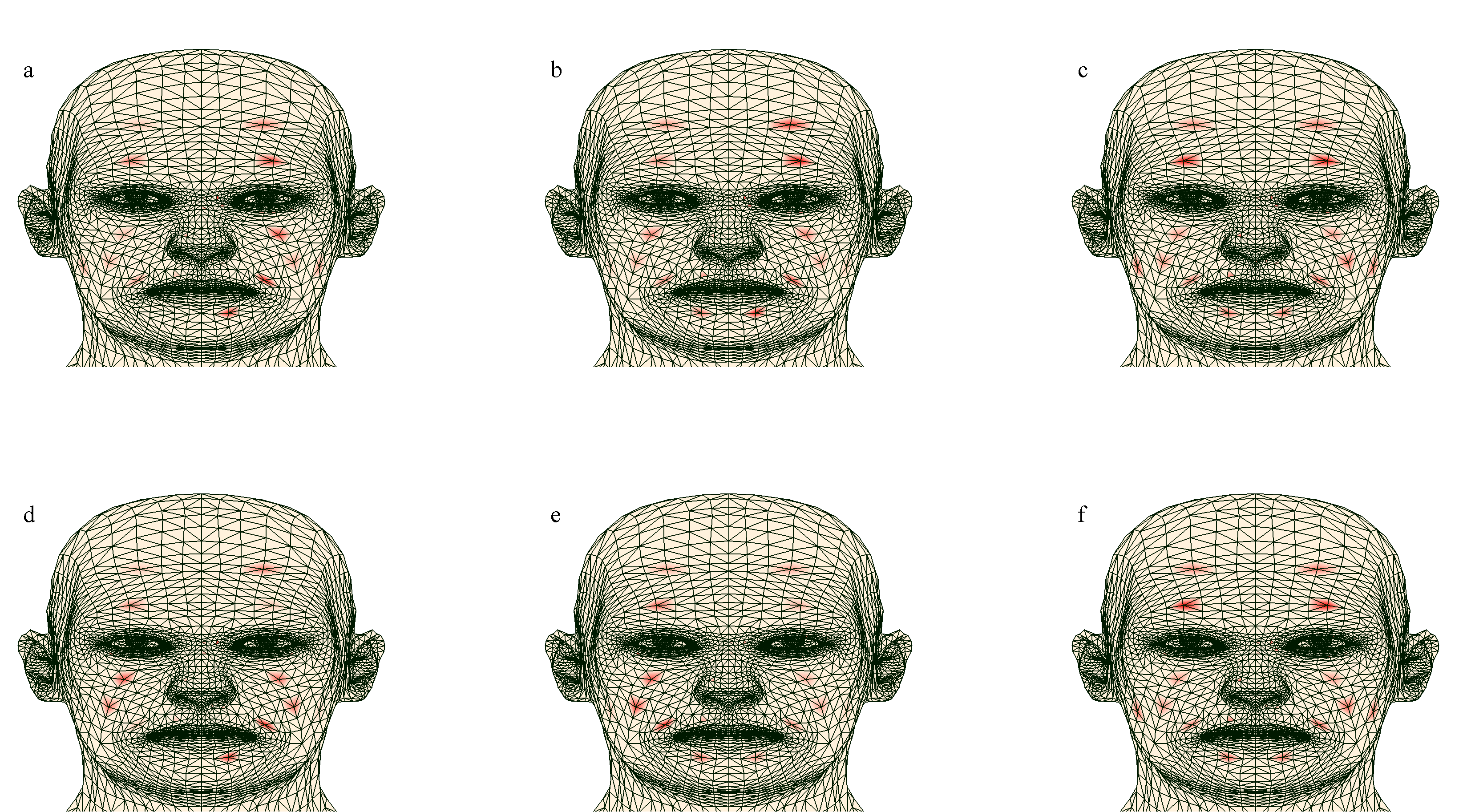


**Figure S9:** Facial muscle activity representations based on the FuzzyEn method for the fear facial expression. A) Estimated muscle activities of patient A before rehabilitation. B) Estimated muscle activities of patient A after rehabilitation. C, F) Mean value of estimated muscle activities of ten healthy individuals. D) Estimated muscle activities of patient B before rehabilitation. E) Estimated muscle activities of patient B after rehabilitation.


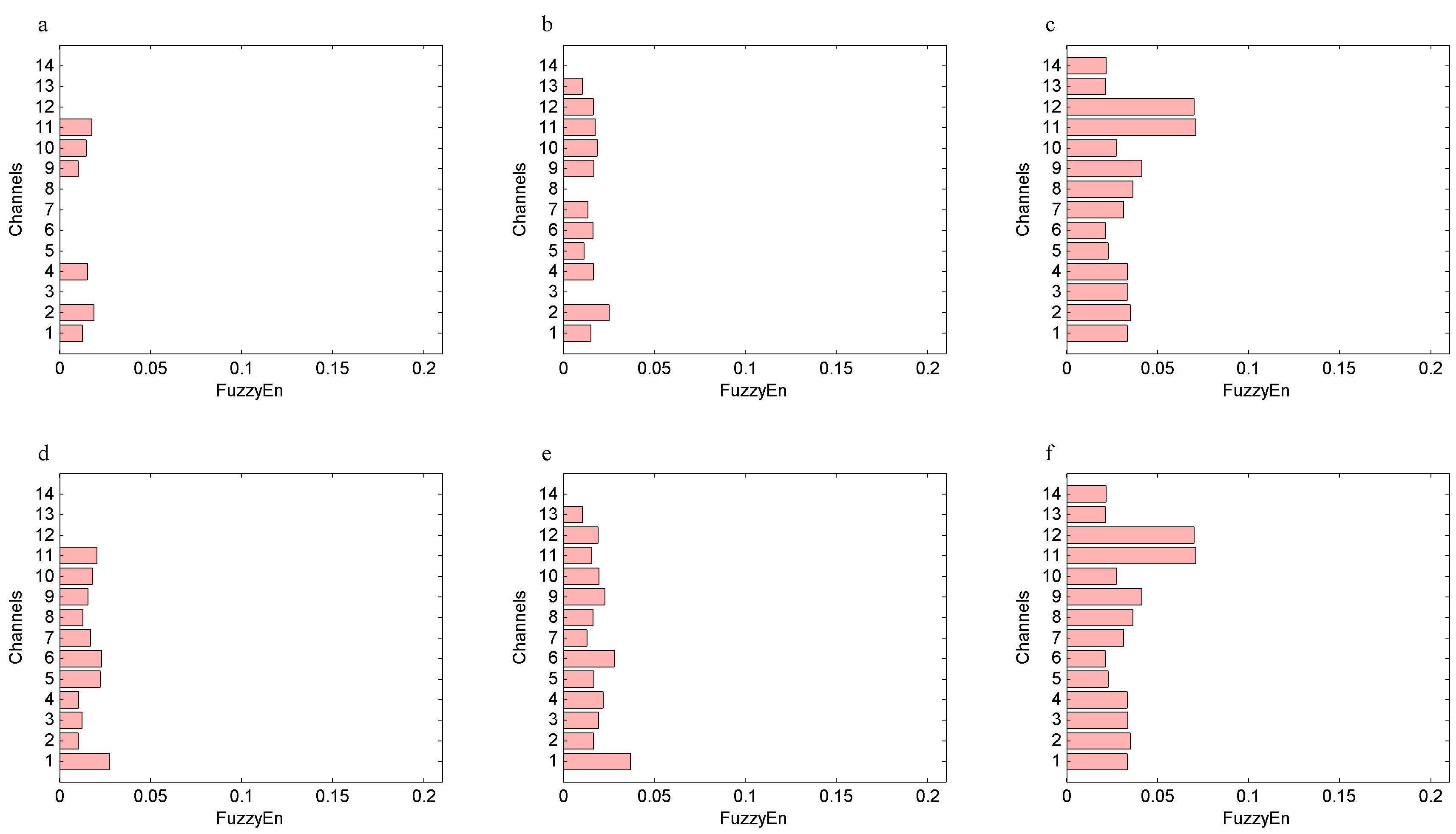


**Figure S10:** Facial muscle activity representations on a 3D face model based on the FuzzyEn method for the fear facial expression. A) Estimated muscle activities of patient A before rehabilitation. B) Estimated muscle activities of patient A after rehabilitation. C, F) Mean value of estimated muscle activities of ten healthy individuals. D) Estimated muscle activities of patient B before rehabilitation. E) Estimated muscle activities of patient B after rehabilitation.


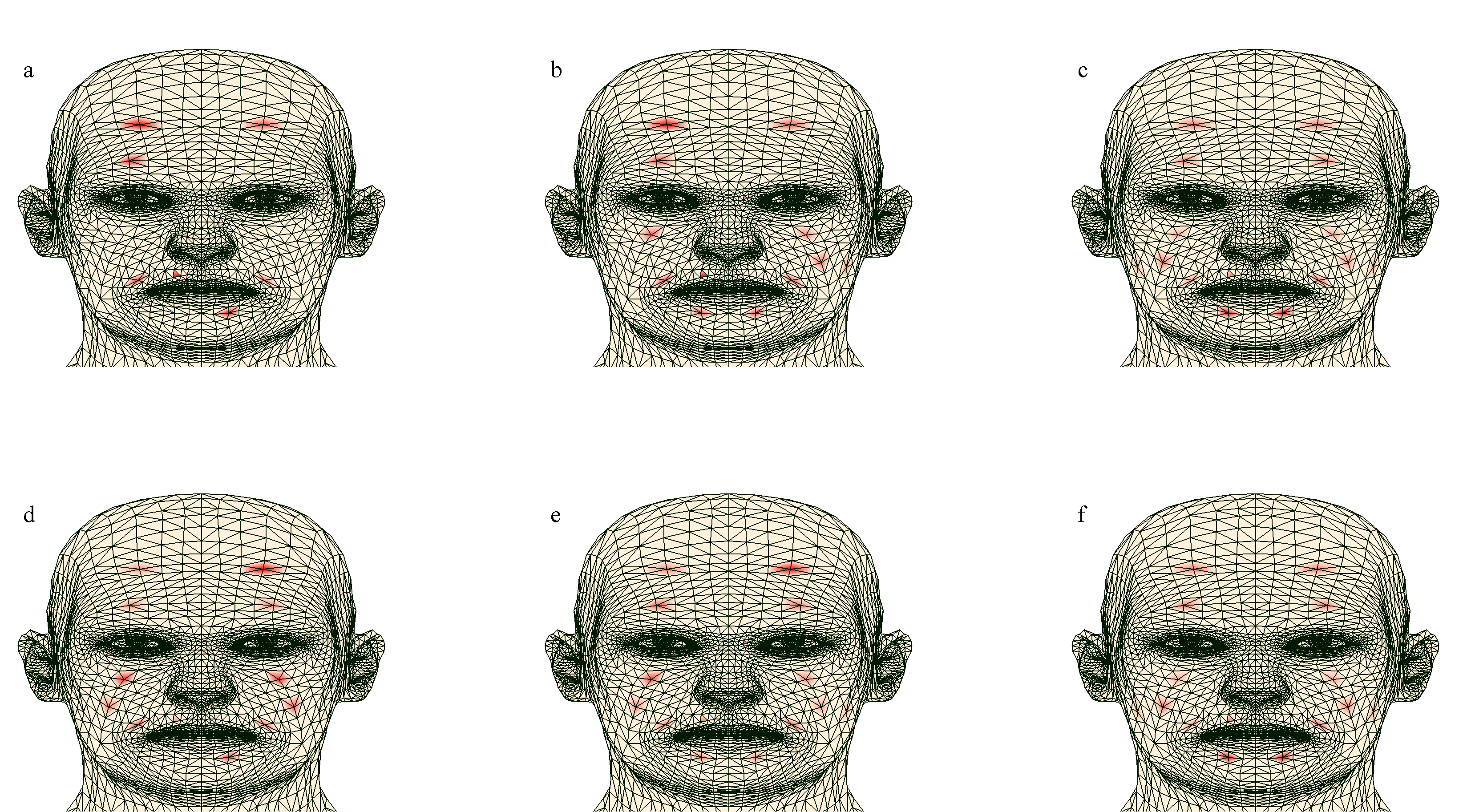


**Figure S11:** Facial muscle activity representations based on the FuzzyEn method for the happiness facial expression. A) Estimated muscle activities of patient A before rehabilitation. B) Estimated muscle activities of patient A after rehabilitation. C, F) Mean value of estimated muscle activities of ten healthy individuals. D) Estimated muscle activities of patient B before rehabilitation. E) Estimated muscle activities of patient B after rehabilitation.


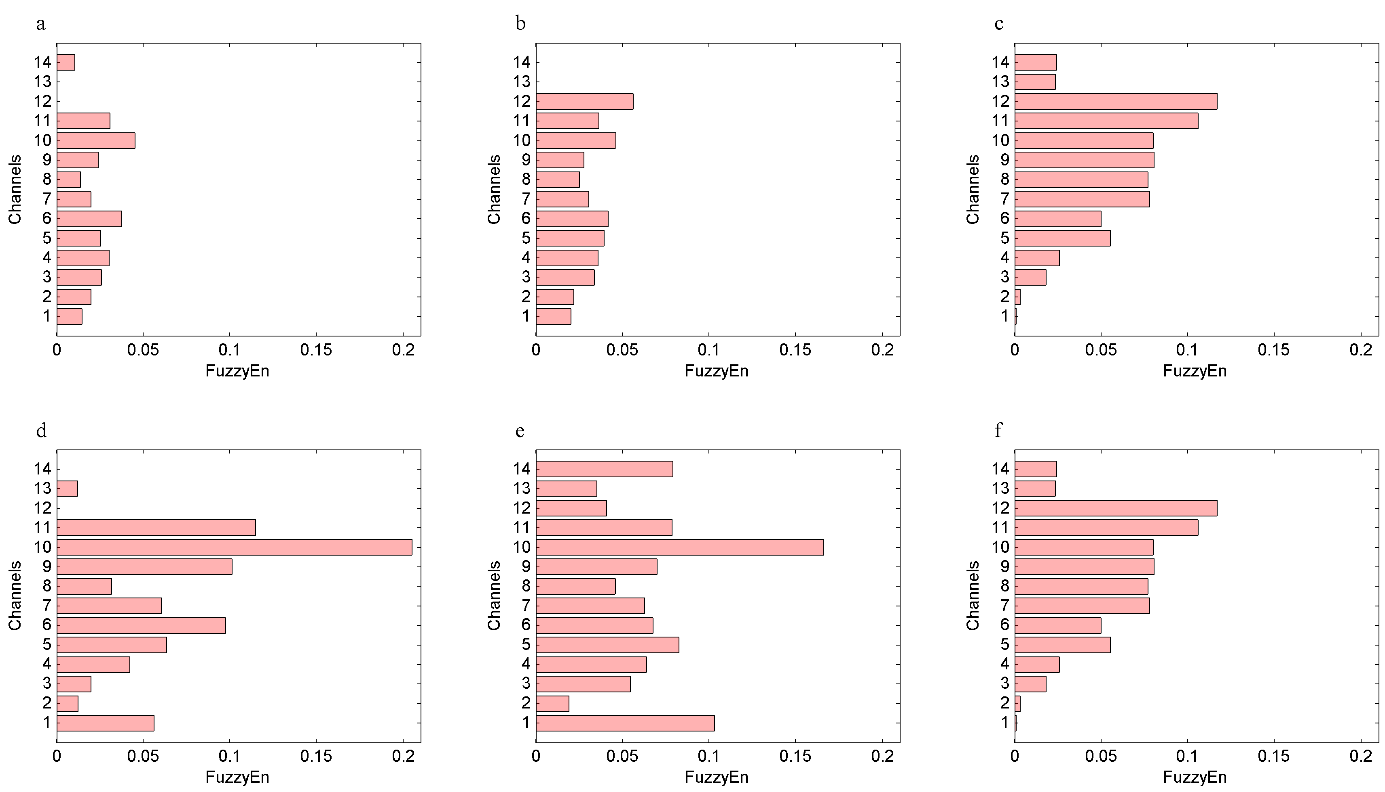


**Figure S12:** Facial muscle activity representations based on the FuzzyEn method for the hate/disgust facial expression. A) Estimated muscle activities of patient A before rehabilitation. B) Estimated muscle activities of patient A after rehabilitation. C, F) Mean value of estimated muscle activities of ten healthy individuals. D) Estimated muscle activities of patient B before rehabilitation. E) Estimated muscle activities of patient B after rehabilitation.


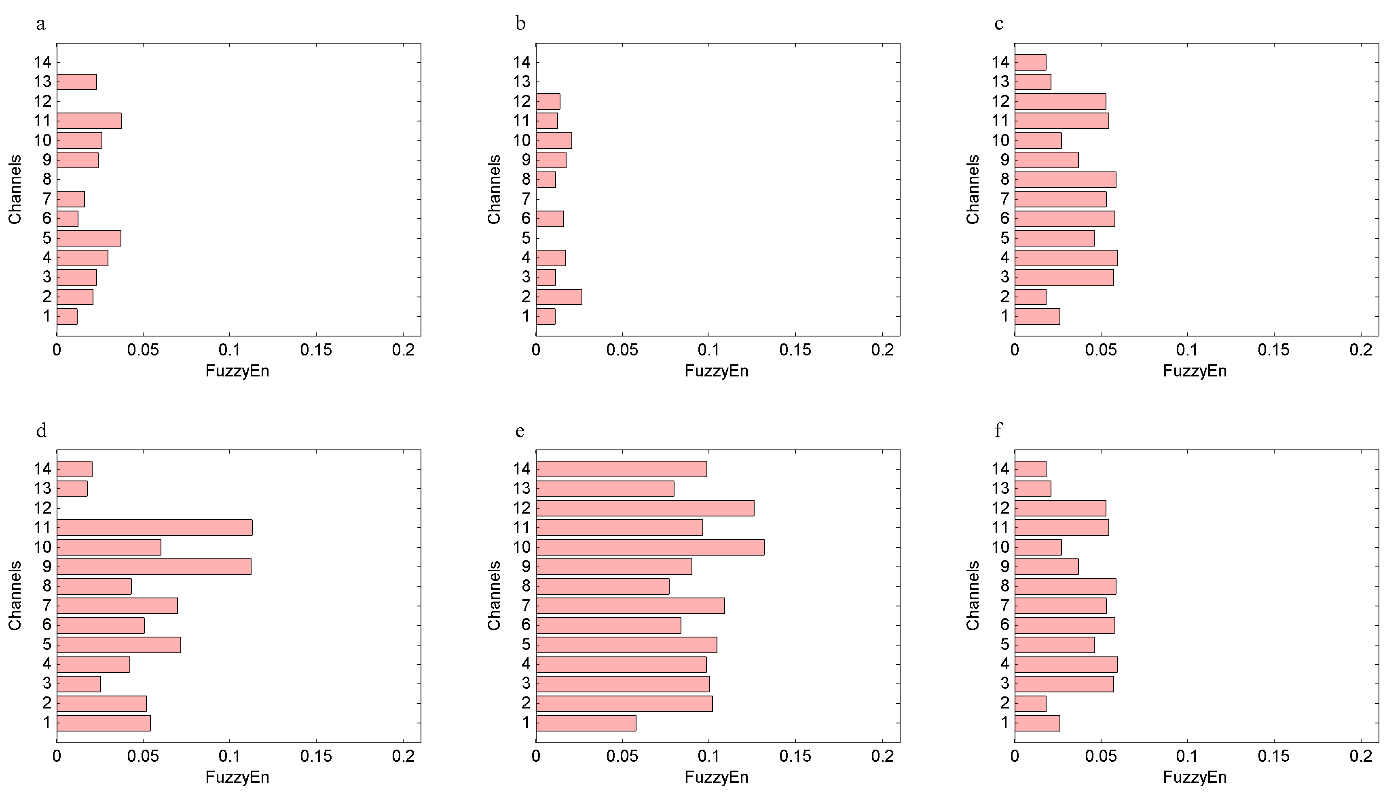


**Figure S13:** Facial muscle activity representations on a 3D face model based on the FuzzyEn method for the hate/disgust facial expression. A) Estimated muscle activities of patient A before rehabilitation. B) Estimated muscle activities of patient A after rehabilitation. C, F) Mean value of estimated muscle activities of ten healthy individuals. D) Estimated muscle activities of patient B before rehabilitation. E) Estimated muscle activities of patient B after rehabilitation.


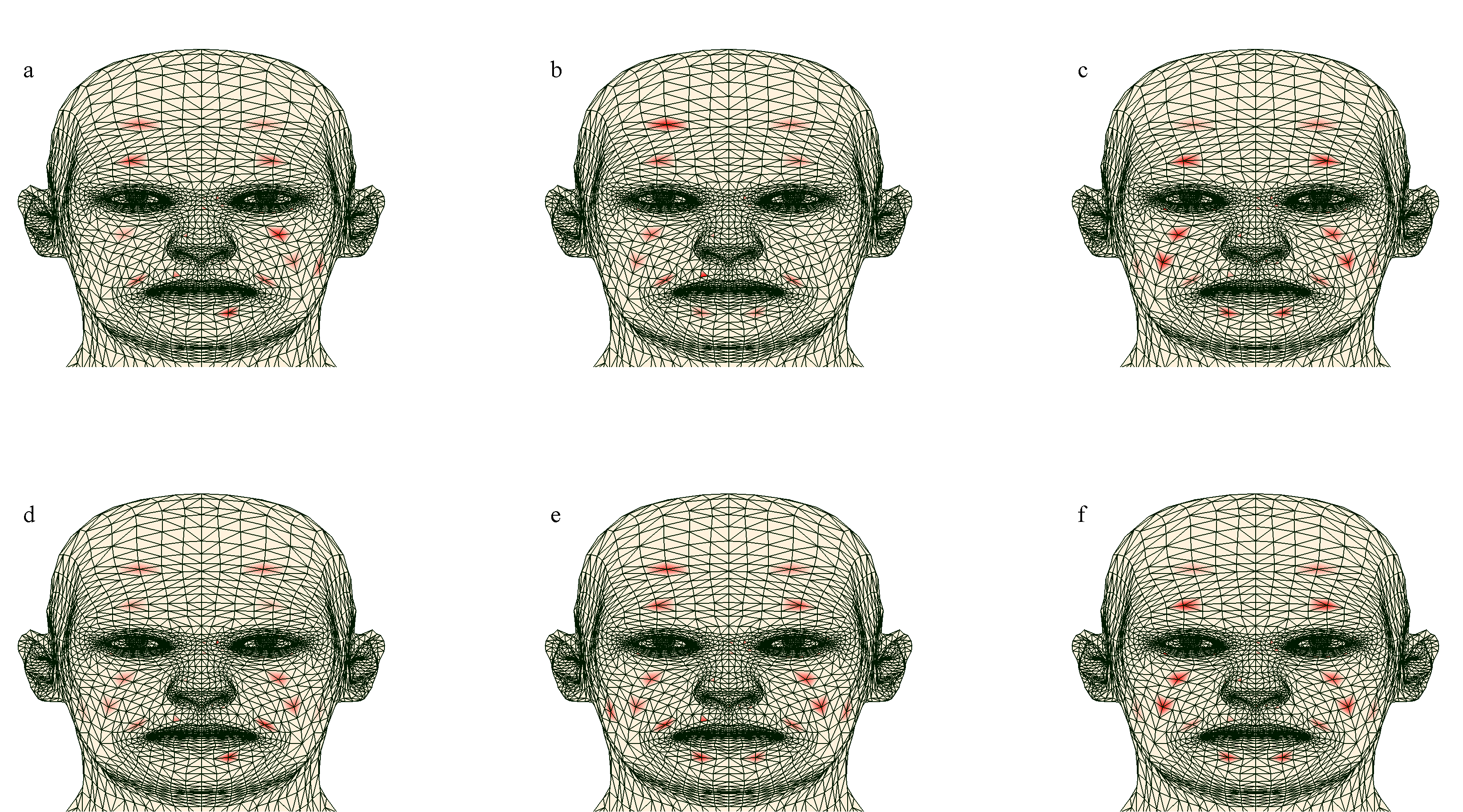


**Figure S14:** Facial muscle activity representations based on the FuzzyEn method for the sadness facial expression. A) Estimated muscle activities of patient A before rehabilitation. B) Estimated muscle activities of patient A after rehabilitation. C, F) Mean value of estimated muscle activities of ten healthy individuals. D) Estimated muscle activities of patient B before rehabilitation. E) Estimated muscle activities of patient B after rehabilitation.


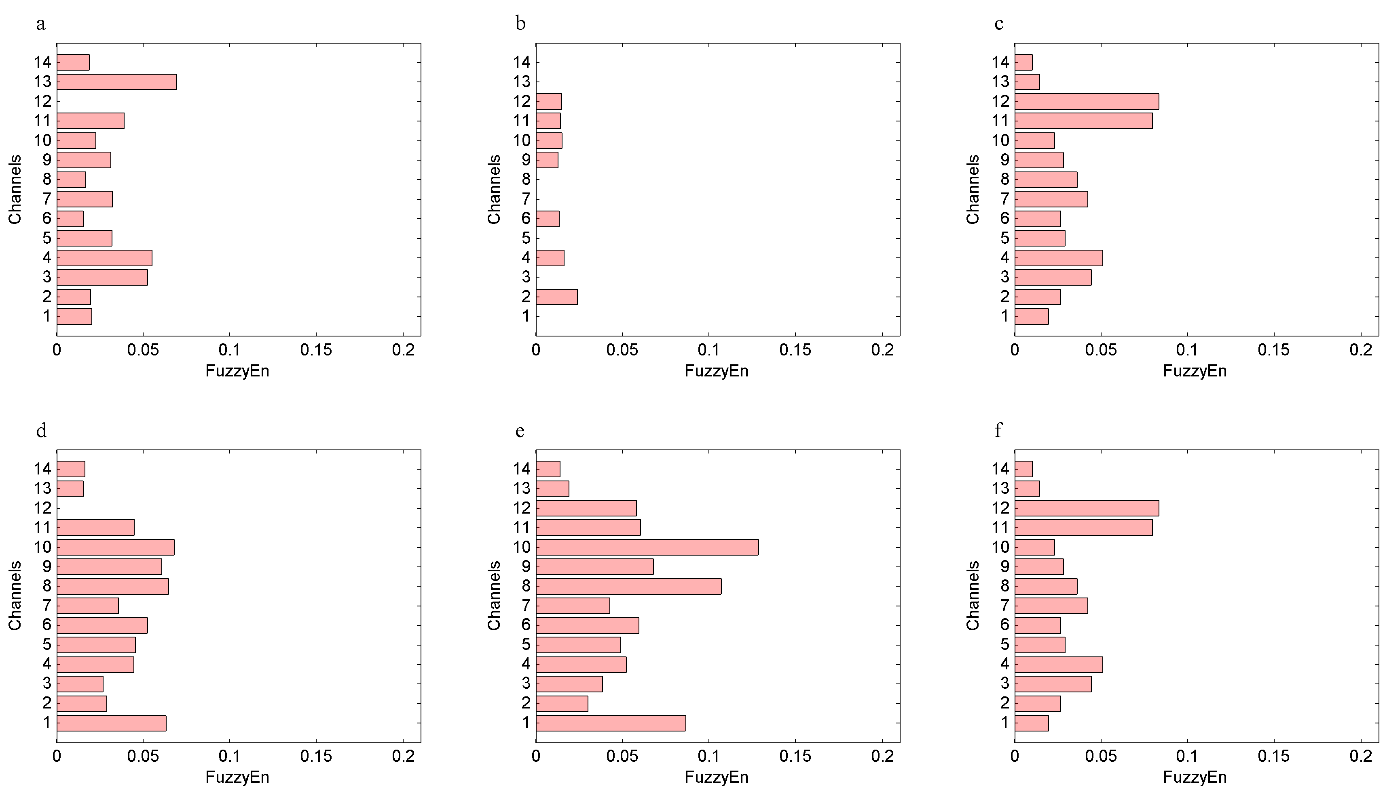


**Figure S15:** Facial muscle activity representations on a 3D face model based on the FuzzyEn method for the sadness facial expression. A) Estimated muscle activities of patient A before rehabilitation. B) Estimated muscle activities of patient A after rehabilitation. C, F) Mean value of estimated muscle activities of ten healthy individuals. D) Estimated muscle activities of patient B before rehabilitation. E) Estimated muscle activities of patient B after rehabilitation.


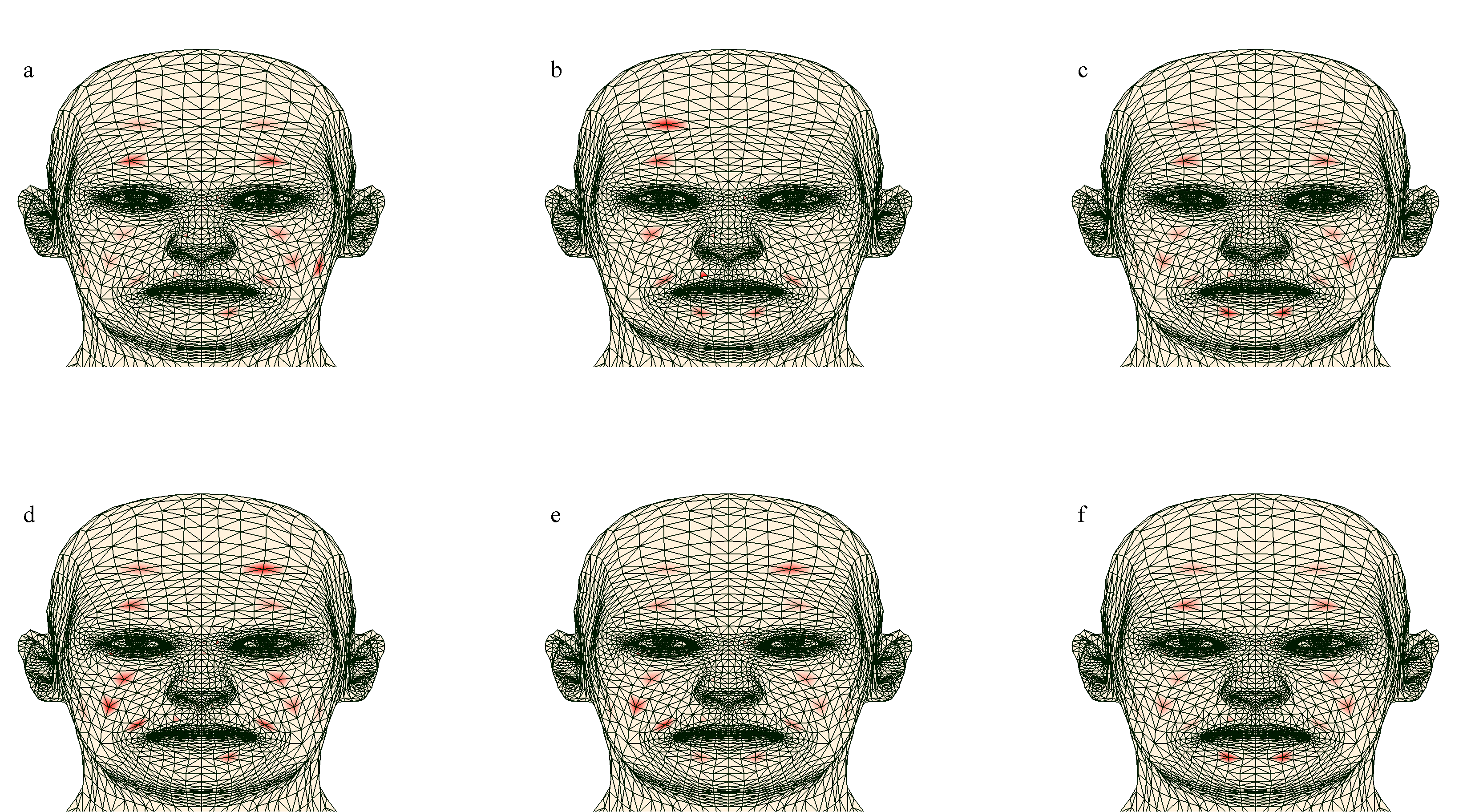


**Figure S16:** Facial muscle activity representations based on the FuzzyEn method for the surprised facial expression. A) Estimated muscle activities of patient A before rehabilitation. B) Estimated muscle activities of patient A after rehabilitation. C, F) Mean value of estimated muscle activities of ten healthy individuals. D) Estimated muscle activities of patient B before rehabilitation. E) Estimated muscle activities of patient B after rehabilitation.


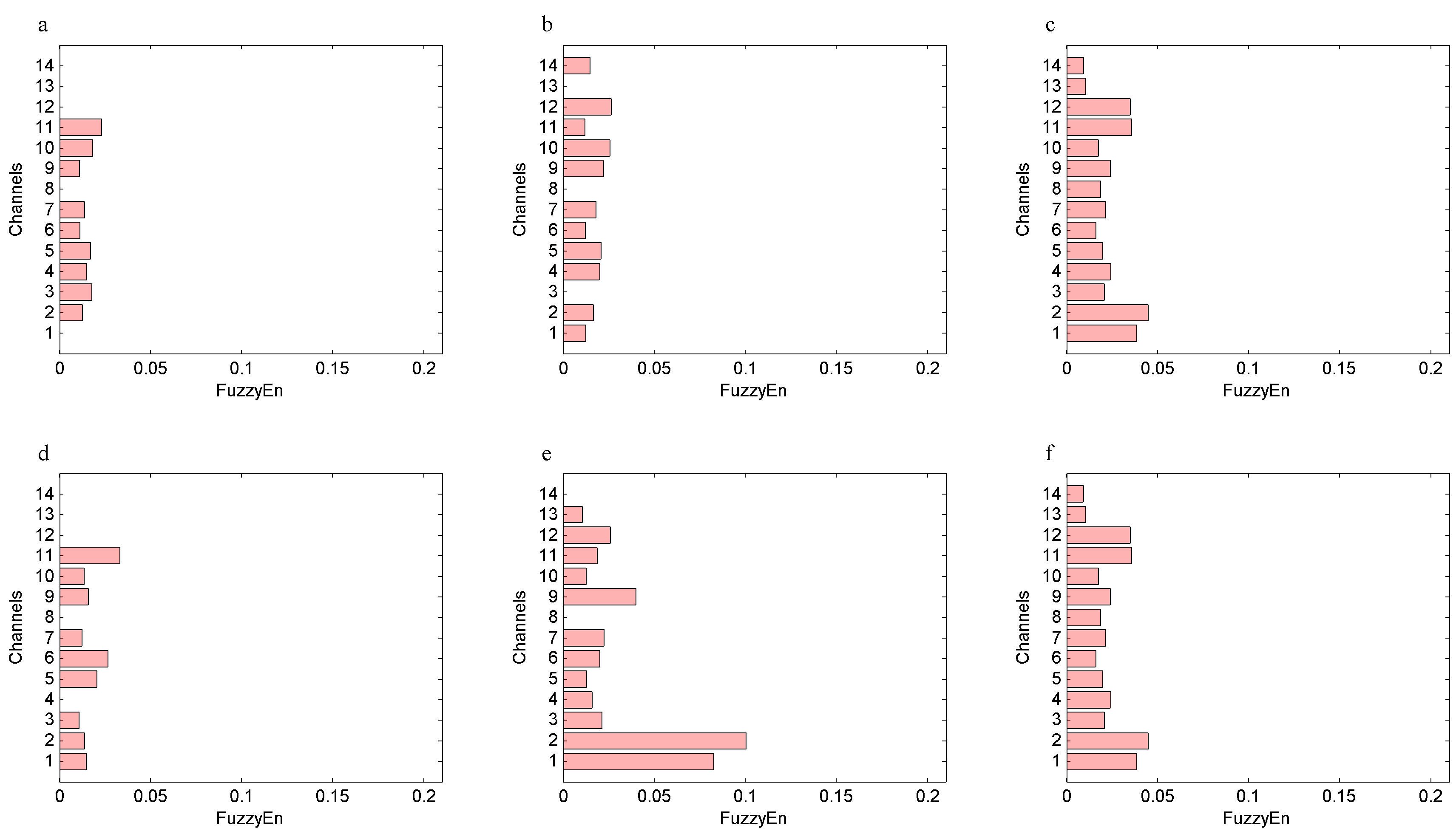


**Figure S17:** Facial muscle activity representations on a 3D face model based on the FuzzyEn method for the surprised facial expression. A) Estimated muscle activities of patient A before rehabilitation. B) Estimated muscle activities of patient A after rehabilitation. C, F) Mean value of estimated muscle activities of ten healthy individuals. D) Estimated muscle activities of patient B before rehabilitation. E) Estimated muscle activities of patient B after rehabilitation.


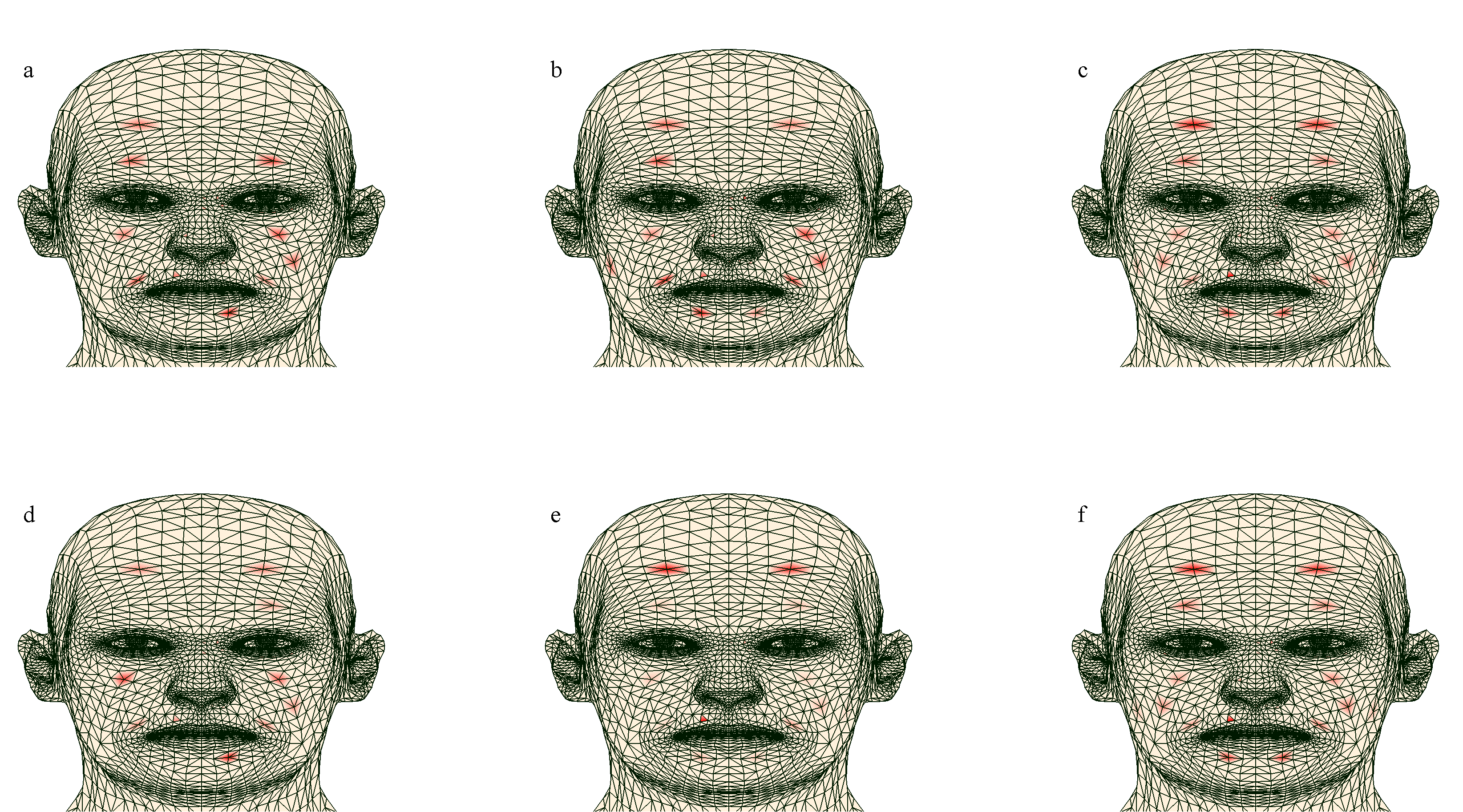


Table S1: Selected functional electrical stimulation channels of patient A during rehabilitation.

| Patient A | channel-1 | channel-2 | channel-3 | channel-4 | channel-5 | channel-6 | channel-7 | channel-8 | channel-9 | channel-10 | channel-11 | channel-12 | channel-13 | channel-14 |
| --- | --- | --- | --- | --- | --- | --- | --- | --- | --- | --- | --- | --- | --- | --- |
| lip funneler |  |  |  |  |  | X |  |  | X |  |  | X | X |  |
| lid tightener |  |  |  |  |  | X | X |  |  |  |  |  | X |  |
| lip puckerer |  |  |  |  |  |  | X | X |  |  |  | X |  |  |
| outer brow raiser | X | X | X | X | X |  | X |  |  |  |  |  |  |  |
| anger | X |  |  |  |  |  | X | X |  |  |  | X |  |  |
| fear |  |  |  |  |  |  | X | X | X |  | X | X | X |  |
| happiness |  |  |  |  | X |  | X | X | X | X | X | X | X |  |
| hateful/disgust |  |  |  |  |  |  | X | X | X |  |  | X | X |  |
| surprised | X |  |  |  |  |  | X | X |  |  |  | X | X |  |
| sadness | X |  |  |  |  |  | X | X |  |  |  | X |  |  |

Table S2: Selected functional electrical stimulation channels of patient B during rehabilitation.

| Patient B | channel-1 | channel-2 | channel-3 | channel-4 | channel-5 | channel-6 | channel-7 | channel-8 | channel-9 | channel-10 | channel-11 | channel-12 | channel-13 | channel-14 |
| --- | --- | --- | --- | --- | --- | --- | --- | --- | --- | --- | --- | --- | --- | --- |
| lip funneler |  |  |  |  |  | X |  | X |  |  | X | X |  |  |
| lid tightener | X | X | X | X |  |  |  |  |  |  |  |  |  |  |
| lip puckerer |  |  |  |  | X | X | X | X |  |  |  |  |  |  |
| outer brow raiser | X | X | X | X |  |  |  |  |  |  |  |  | X |  |
| anger | X | X | X | X |  |  |  |  |  |  |  |  | X |  |
| fear | X | X |  |  |  |  |  |  | X |  |  |  |  |  |
| happiness |  |  |  |  |  |  | X | X |  |  | X | X |  |  |
| hateful/disgust |  | X |  |  |  |  | X | X |  |  |  |  | X |  |
| surprised | X | X |  | X |  |  | X | X |  |  | X |  |  | X |
| sadness |  | X |  |  |  |  | X | X |  |  |  |  |  |  |

Table S3: Summary of data used.

| Code | Age | Sex | Height | Weight |
| --- | --- | --- | --- | --- |
| V01 | 35 | m | 180 | 82 |
| V02 | 36 | m | 189 | 102 |
| V03 | 35 | m | 182 | 81 |
| V04 | 35 | m | 174 | 65 |
| V05 | 35 | m | 185 | 82 |
| V06 | 30 | m | 178 | 83 |
| V07 | 29 | m | 170 | 60 |
| V08 | 27 | m | 182 | 112 |
| V09 | 24 | m | 170 | 75 |
| V10 | 21 | m | 178 | 87 |
|  | Average age: 31 | Total number: 10 | | |

Table S4: Semmes-Weinstein’s monofilament test results for patient A at 19 and 39 months after surgery.

| Patient A | **Touch (19 months)**  **(After surgery)** | | **Touch (39 months)**  **(After surgery)** | |
| --- | --- | --- | --- | --- |
|  | Right | Left | Right | Left |
| Forehead | 2.83 | 2.83 | 3.61 | 3.61 |
| Above Eyebrows | 2.83 | 2.83 | 3.61 | 3.61 |
| Eyelid | 2.83 | 2.83 | 3.61 | 3.61 |
| Eyebrow midpoint | 2.83 | ND | 4.31 | ND |
| Nose | 2.83 | 2.83 | 4.31 | 4.31 |
| Upper lip | 2.83 | ND | 4.31 | ND |
| Lower lip | 2.83 | ND | 4.31 | ND |
| Chin | 2.83 | 3.61 | 4.56 | 4.56 |
| Below Ear | 3.61 | 3.61 | 4.56 | 4.56 |
| Cheek | 2.83 | 2.83 | 4.31 | 4.31 |

(ND: Not determined)

Table S5: Semmes-Weinstein’s monofilament test results for patient B at 23 and 43 months after surgery.

| Patient B | **Touch (23 months)**  **(After surgery)** | | **Touch (43 months)**  **(After surgery)** | |
| --- | --- | --- | --- | --- |
|  | Right | Left | Right | Left |
| Forehead | 2.83 | 2.83 | 3.61 | 3.61 |
| Above Eyebrows | 3.61 | 3.61 | 3.61 | 3.61 |
| Eyelid | 3.61 | 3.61 | 4.31 | 4.31 |
| Eyebrow midpoint | 3.61 | ND | 3.61 | ND |
| Nose | 2.83 | 2.83 | 3.61 | 3.61 |
| Upper lip | 3.61 | ND | 4.31 | ND |
| Lower lip | 3.61 | ND | 4.31 | ND |
| Chin | 3.61 | 3.61 | 4.31 | 4.31 |
| Below Ear | ND | ND | 4.31 | 4.56 |
| Cheek | 2.83 | 2.83 | 4.31 | 4.56 |

(ND: Not determined)
